# Unveiling the burden of COPD: perspectives on a patient-reported outcome measure to support communication in outpatient consultations—an interview study among patients

**DOI:** 10.3389/fresc.2024.1434298

**Published:** 2024-09-02

**Authors:** Louise Muxoll Gronhaug, Ingeborg Farver-Vestergaard, Jannie Christina Frølund, Cecilie Lindström Egholm, Anders Løkke Ottesen

**Affiliations:** ^1^ Department of Medicine, Vejle Hospital, Lillebaelt Hospital, University Hospital of Southern Denmark, Vejle, Denmark; ^2^ Department of Regional Health Research, University of Southern Denmark, Odense, Denmark; ^3^ REHPA, The Danish Knowledge Centre for Rehabilitation and Palliative Care, Odense University Hospital, Nyborg, Denmark; ^4^ Department of Clinical Research, University of Southern Denmark, Odense, Denmark

**Keywords:** PROM, chronic obstructive pulmonary disease, person-centered, patient-centered, holistic, palliative, qualitative content analysis, EORTC

## Abstract

**Introduction:**

Chronic Obstructive Pulmonary Disease (COPD) profoundly affects physical, psychological, and social aspects of life, yet these issues often remain unaddressed. Patient-Reported Outcomes Measures (PROM) have the potential to address these issues by promoting person-centered communication. However, their impact in COPD practice remains uncertain. This study aimed to investigate how patients with COPD perceive the usefulness of a new holistic PROM for general palliative care (PRO-Pall) before and during outpatient consultations.

**Methods:**

Semi-structured telephone interviews were conducted with patients diagnosed with moderate to very severe COPD, 2-5 days after consultation at a respiratory outpatient clinic in Denmark. Interviews were transcribed verbatim and analyzed inductively using qualitative content analysis.

**Results:**

Nine patients (five males; mean age: 66 years) participated in the study with four themes emerging: (1) Unlocking thoughts: Completing PRO-Pall stimulated patients’ self-reflection, which revealed previously overlooked COPD-related issues, particularly psychosocial challenges. (2) Unmasking concerns: Patients felt encouraged to be honest, rather than concealing their concerns. (3) Breaking the ice: PRO-Pall responses enabled direct questioning by healthcare professionals during consultations, initiating discussions on patients’ sensitive yet vital COPD-related matters. (4) Deepening the dialogue: Healthcare professionals’ targeted and attentive approach fostered more holistic and meaningful discussions, providing most patients with a deeper understanding of psychosocial issues affecting their well-being.

**Conclusion:**

Completing PRO-Pall prior to outpatient consultations prompted most patients with COPD to unveil previously unacknowledged psychosocial challenges. During consultations, addressing these challenges initiated open discussions on individual concerns, enhancing most patients’ understanding of the multifaceted burden of COPD.

## 1 Background

Patients with Chronic Obstructive Pulmonary Disease (COPD) encounter multiple challenges beyond the progression of physical symptoms like dyspnea and coughing (1, 2). These challenges include fatigue, psychological distress, and social isolation, all of which profoundly impact patients’ daily life, prognosis, as well as quality of life and well-being (3–6). Given the unpredictable trajectory of COPD, it is crucial for healthcare professionals (HCPs) to recognize its diverse impact on patients (7).

Unmet support needs are common among patients with advanced COPD (8). For example, psychological distress including symptoms of anxiety and depression often remains unidentified and untreated, adversely impacting patients’ well-being (4). Therefore, solely focusing on improving patients’ functional life, such as symptom reduction, as emphasized in patient-centered care, is inadequate. Instead, a person-centered approach becomes essential to enhance patients’ subjective experience of well-being and promote a meaningful life (9). Holistic communication between patients and HCPs plays a pivotal role in promoting well-being and requires that HCPs consider the entirety of the individual beyond the illness (10, 11). Communication must be tailored to address patients’ individual challenges and support needs (6, 8), across physical, psychological, social and existential aspects of their lives (8, 12, 13). However, this requires that the HPCs have routine access to patients’ issues and challenges (3, 14, 15).

Patient-reported Outcome Measures (PROMs) are increasingly integrated in routine practices worldwide to access patients’ perspectives (16, 17). PROMs, standardized questionnaires designed to collect information directly from patients, encompass data on health status, symptoms, and health-related quality of life (18). They hold the potential to promote patient engagement (19–23), improve communication and shared decision-making (17, 24), and support patients’ disease management, thereby enhancing quality of life (23–26).

In COPD practice, PROMs have traditionally been used in clinical trials to assess disease severity, health status, and treatment effectiveness. These assessments primarily focus on evaluating the impact on physical symptoms, exercise capacity and activities of daily living (27–30). Integrating PROMs into rehabilitation programs may promote patients’ understanding, management of COPD and well-being (31). However, utilization of holistic PROMs are required to promote person-centered care (23, 32).

In Denmark, a holistic “PROM for general palliative care” (PRO-Pall) has been developed to assess patients’ perspectives on physical, psychosocial, and existential aspects of everyday life affected by chronic or life-threatening illnesses to promote comprehensive communication between patients and HCPs across various settings, including respiratory clinics (33). Applying this instrument is based on the notion that basic palliative care should start early in the COPD illness trajectory, as the course of COPD is more unpredictable than the course of other illnesses, such as cancer, and should not be confused with end-of-life care (34). The Danish Health Data Authority led the development of PRO-Pall, which was based on a political initiative to leverage the potential positive effects of using PROMs. Utilization of PRO-Pall underwent feasibility testing in 2022 and was nationally launched for clinical practice in 2023 (35).

Despite their potential, the impact of integrating PROMs, including PRO-Pall, into routine COPD practice remains unclear, particularly in facilitating person-centered communication from the patients’ perspectives. Addressing these uncertainties is pivotal for improving COPD care strategies. For these reasons, the objectives of this interview study were to investigate how patients with COPD experienced and perceived the usefulness of PRO-Pall utilization (1) before and (2) during consultations at a respiratory outpatient clinic.

We addressed the following research questions:

- How does completing PRO-Pall before consultations prepare patients to engage in discussions about their COPD-related issues and concerns with HCPs?
- How are PRO-Pall responses utilized during consultations, and how does it contribute to discussing patients’ individual COPD-related issues and concerns?

## 2 Methods and materials

We employed an inductive, interpretive approach in this qualitative study, emphasizing hermeneutic principles. We recognized the central and active role of the researcher throughout the research process, including the concept of co-creating data with study participants (36–38).

### 2.1 Setting and administration of the PRO-Pall questionnaire

**Figure 1:**
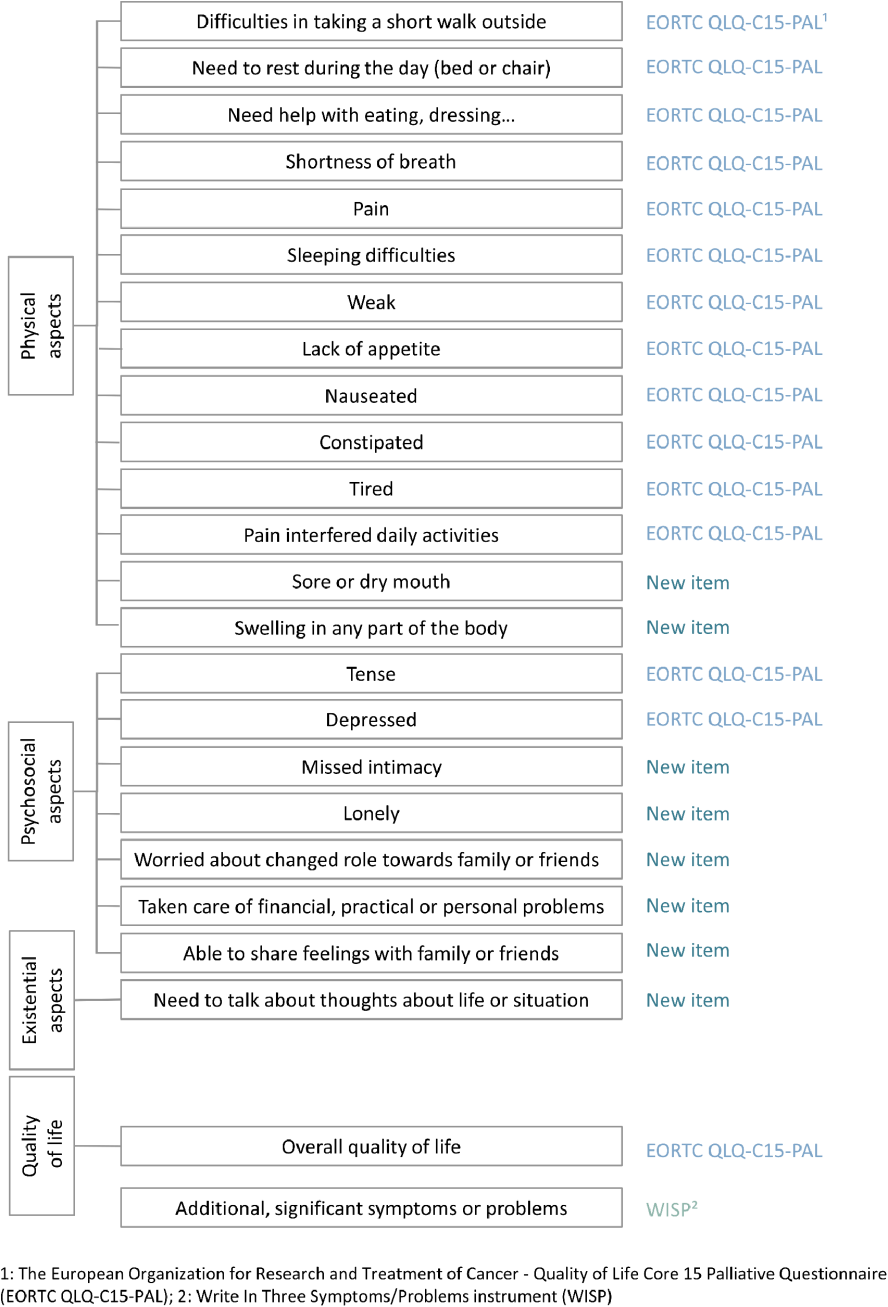
Items in PROM for general palliative care (PRO-Pall).

The participants in this interview study were patients with COPD who attended clinical consultations at the respiratory outpatient clinic of Vejle Hospital in Denmark after completing PRO-Pall, which consists of 24 items (33). Eight new items were specifically developed to supplement “The European Organization for Research and Treatment of Cancer—Quality of Life Core 15 Palliative Questionnaire” (EORTC QLQ-C15-PAL) (39) and “Write In Three Symptoms/Problems” (WISP) (40). Most items are answered by indicating how patients have experienced or felt various issues during the last week, using ratings from 1 (not at all) to 4 (very much) (39, 33). Figure 1 presents the specific content of each item, illustrating the respective PROM it belongs to and whether it addresses physical, psychosocial, or existential aspects.

A dedicated PRO-Pall team, with two experienced physicians (respiratory specialists) and one specialized nurse, who also served as the PRO-Pall coordinator, was tasked with integrating PRO-Pall into consultations among patients with COPD. Initially, designated “PRO-Pall days” were planned, allowing for one-hour consultations to facilitate the team's familiarity with utilizing PRO-Pall responses to support holistic conversations with the patients. This hands-on experience enabled the team to gain practical insights into effectively integrating PRO-Pall into patient consultations. While there was no formal additional training, the team continuously discussed their experiences with each other to refine their approach. After approximately one month, the team reverted to routine practice, allocating 30–45 min per patient for consultations. This allowed for the integration of “PRO-Pall consultations” alongside “standard” consultations, resulting in an average of around 20 PRO-Pall consultations per month.

Approximately one month prior to their scheduled “PRO-Pall consultations”, patients received written instructions to complete PRO-Pall online. The secure “My Hospital” app was utilized to distribute PRO-Pall to the patients, allowing them to respond via smartphone, tablet, or computer. Additionally, this platform was used to collection and storage of PRO-Pall data, integrating it into each patient's medical record. The PRO-Pall team had access to patients’ responses both before and during consultations. Initially, patients engaged in discussions with the specialized PRO-Pall nurse during consultations, followed by subsequent discussions with one of the two PRO-Pall physicians.

### 2.2 Sampling

The inclusion criteria for the purpose of the present interview study were: (1) Patients diagnosed with moderate to very severe COPD (1), (2) age ≥ 40 years, (3) having received PRO-Pall before consultation. To gain in-depth and nuanced data, our exclusion criteria were: (1) Inability to complete PRO-Pall at home or upon arrival at the clinic, (2) inability to comprehend or express themselves in Danish due to language barriers or cognitive impairment, and (3) hospitalization within the last month. The latter criterion was defined to ensure that our sample represents the stable outpatient population, as typical patients in outpatient settings would not have been recently hospitalized.

The PRO-Pall coordinator employed purposive sampling (41, 42) to recruit eligible patients from the respiratory clinic and scheduled a telephone interview at a time convenient for the patient if they consented to participate. During the recruitment phase, the PROPall coordinator maintained ongoing communication with the interviewer to ensure sufficient diversity in sex, age, and COPD severity within the sample. We anticipated to recruit approximately 10 participants to gather adequate and nuanced data to answer the research questions (43). Preliminary analysis was initiated after the fifth interview and continued after each subsequent interview until the ninth interview, at which time sufficient information power was deemed adequate, thereby strengthening the trustworthiness of the findings (42, 44).

### 2.3 Data collection

The patients’ experiences and perceptions were investigated through individual semi-structured interviews (43). The interviewer contacted all participants by phone at the scheduled time and offered them the option of a face-to-face interview at home or at the hospital. However, the participants preferred telephone interviews. This method, noted for its cost effectiveness, is particularly suitable when interviewing elderly and fragile individuals, such as those with COPD (45), about sensitive issues (46, 47). Two of the patients’ relatives were present during the interview but did not participate.

Following international recommendations, we developed a semi-structured interview guide in alignment with the study objectives and based on previously presented literature (48), including national experiences with PRO-Pall development (49). The interview guide, accessible in the Supplementary Material, consisted of direct, open-ended questions aimed at eliciting detailed descriptions of patients’ subjective experiences and perceptions (43, 48). The main themes covered were: (1) completing PRO-Pall as a preparation before consultation, and (2) utilizing PRO-Pall responses to discuss individual issues during consultations. The questions for each theme aimed to explore the patients’ perspectives on (a) observable aspects and concrete information, (b) experiences and emotions during the event, and (c) interpretation and evaluation of the event (50). Given that patients with COPD generally have limited health literacy (51), questions were crafted using straightforward language with a LIX readability index of 28 (easy to read).

Refinements to the guide were made based on the interviewer's clinical observations and discussions with the PRO-Pall team. The interview guide underwent pilot testing with two participants to assess its relevance and feasibility. After confirming the guide's relevance and determining that no significant adjustments were needed, the two pilot interviews were included in the final dataset.

Interviews were conducted 2–5 days following the consultation to minimize the risk of recall bias. To ensure consistency throughout the data collection, all interviews were conducted by the same researcher (43). The interviewer (first author) was a PhD student and a former clinical nurse specialist (RN, MScN) with some expertise in conducting interviews. Prior to the data collection the interviewer had observed four PRO-Pall consultations to gain insights into the utilization of PRO-Pall responses. The interviewer had no prior contact with the participants, but introduced herself as a research nurse (PhD student) at the beginning of each interview.

The semi-structured interview format provided flexibility, enabling a nuanced exploration of participants’ responses to generate richer data. The interviewer explored additional aspects of PRO-Pall utilization by employing interpretive, follow-up, and probing questions, coupled with moments of silence, to prompt participants to recall, reflect, and elaborate on their experiences. After each interview, the interviewer took field notes to document her immediate impressions, including cues and any notable aspects of the interview (43, 53).

### 2.4 Data analysis

The interviews were audio-recorded and transcribed verbatim by the interviewer. After each interview, the audio recordings were reviewed while the interviewer simultaneously read the transcriptions to ensure accuracy. The transcripts and field notes were inductively analyzed using Qualitative Content Analysis (QCA) (52, 54). The QCA method is frequently applied in health research and is known for promoting transparency and fostering a nuanced understanding of participants’ experiences and perspectives in specific contents. QCA is particularly valuable for generating novel insights directly applicable to improving healthcare (55, 56).

The approach was inductive, with themes derived from the data through an iterative process, including four steps: (1) establishing an overview of the material, (2) identifying and extracting meaning units, (3) condensing meaning units into descriptive categories, and (4) generating explanatory themes (54, 36). The preliminary analysis (step 1 and 3) was conducted after the fifth interview and was continued after each subsequent interview to assess when sufficient data were gathered to address the study objectives (42).

NVivo software was utilized for coding, extracting, and clustering the meaning units, thereby enabling the distinction of similarities and differences and revealing relationships across data, including between sub-categories (57). Gradually, descriptions of the categories evolved with increasing levels of abstraction and interpretation, leading to the final themes. The coding and initial analysis were conducted by the first author, with contributions from the second and third authors in the form of clarifying comments and discussions. The final themes and the conceptual model were also discussed with the other coauthors and the PRO-Pall coordinator to ensure consensus. The analytic steps supported by NVivo, and the generation of themes documented in a coding tree, is available in the Supplementary Material (2. The analytic steps and coding tree). Patient quotations, identified codes, categories, and themes were linguistic analyzed and translated into English for publication (58).

### 2.5 Ethical considerations

The study adhered to tenets of the Declaration of Helsinki (59) and the Danish Code of Conduct for Research Integrity (60). As per Danish law, qualitative studies do not require notification to the Regional Committee on Health Research Ethics. However, approval for data management and storage was obtained from the Danish Data Protection Agency in the Region of Southern Denmark (No. 22/993). Confidentiality and participants’ integrity were protected through the use of pseudonyms in all documents. All transcripts were securely stored in NVivo and will be destroyed when no longer needed.

Before they provided written consent, the participants were thoroughly informed about the study's purpose, voluntary participation, and the option to withdraw. None of the participants utilized the opportunity to contact the interviewer for any queries or concerns before or after the interview. At the beginning of each interview, the participants were asked if they still wished to participate, and the study's aims and objectives were briefly reiterated. Throughout the interviews, the interviewer maintained an open and attentive approach to sense any emotional distress encountered by the participants.

## 3 Results

The following sections present the participant profile and the main findings of the study. Interviews were conducted from September to October 2022, with an average interview duration of 30 min (ranging from 20 to 45 min), excluding the introduction.

### 3.1 Participant characteristics

Of the 13 patients invited to participate in the study, three declined due to lack of capacity or interest, and one dropped out, as she could not be reached by phone. Therefore, nine patients were interviewed. These participants comprised five males and four females with COPD ranging from moderate to very severe, a mean age of 66 years (range: 46–76), and a mean self-reported duration since COPD diagnosis of 13 years (range: 5–20). The interviews took place 2–5 days after the consultations. None of the patients had been hospitalized in the year leading up to the interview. Due to a technical problem, one of the nine patients completed the PRO-Pall upon arrival at the respiratory clinic, resulting in only her experiences during the consultation being included. The participant characteristics are summarized in Table 1.

**Table 1:** The participants’ (*N* = 9) main characteristics in the interview study on PRO-Pall utilization.

ID	Sex	Age groups (years)	Education length^a^	COPD severity	Living situation	Relatives present during consultation
P1	Male	<50	No education	Moderate	Lives with partner	No
P2	Male	50–69	Short	Moderate	Lives alone	No
P3	Male	>70	Medium	Moderate	Lives alone	Yes
P4	Male	>70	Short	Severe	Lives with partner	Yes
P5	Female	50–69	Short	Severe	Lives with partner	No
P6	Female	>70	Medium	Very severe	Lives with partner	Yes
P7	Female	50–69	No education	Severe	Lives alone	No
P8	Male	>70	Long	Severe	Lives with partner	Yes
P9	Female	<50	No education	Moderate	Lives alone	No

^a^ Education length: Short (professional academy programs, typically lasting 2–2½ years), medium (professional bachelor's programs with a duration of 3–4 years), and long (university studies spanning 5–6 years or more).

### 3.2 Main findings

From the extracted meaning units, 22 sub-categories were categorized across eight categories and four final themes were generated:

Objective 1: Completing PRO-Pall before consultations

- Theme 1: Unlocking thoughts
- Theme 2: Unmasking concerns

Objective 2: Discussing PRO-Pall responses during consultations

- Theme 3: Breaking the ice
- Theme 4: Deepening the dialogue

**Figure 2:**
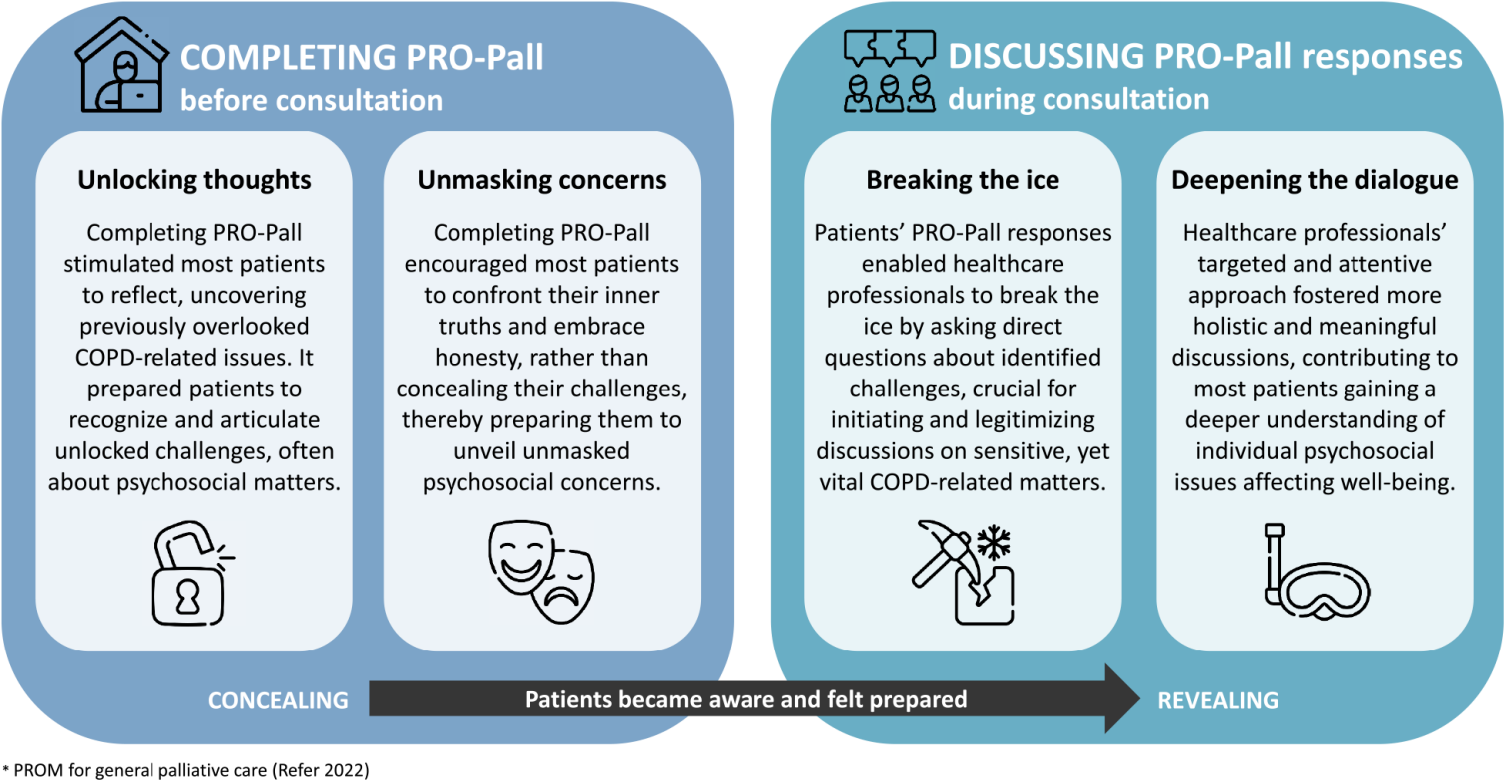
Conceptual model of patient-perceived usefulness of PRO-Pall in COPD consultations.

Based on the four themes, we developed a conceptual model of patient-perceived usefulness of PRO-Pall in COPD consultations. The model are visually represented in Figure 2, with each theme accompanied by a brief description and an icon to illustrate key concepts.

Overall, the patients perceived PRO-Pall as crucial in uncovering and acknowledging their current challenges and concerns most affected by COPD. Patients were accustomed to discuss physical issues with HCPs; however, most patients experienced that the utilization of PRO-Pall responses by HCPs was essential for initiating and targeting discussions on individual psychosocial matters, many of which the patients had never previously discussed with HCPs or relatives. None of the patients mentioned that existential aspects were discussed, nor did they indicate a need for such discussions. Notably, we found no differences within the identified themes related to sex, age, or COPD severity. All the patients emphasized the relevance of PRO-Pall and expressed a hope for its integration into future routine outpatient practice. The themes are described in detail below, supported by participant quotations followed by the participant identifier (e.g., P1) to ensure transparent interpretation and empirical representation.

#### 3.2.1 Theme 1. Unlocking thoughts: completing PRO-Pall stimulated patients to reflect and prepared them to articulate recognized challenges and concerns

Completing PRO-Pall prior to the outpatient consultation stimulated most patients to reflect on the physical and psychosocial aspects affecting their well-being. This reflective process often enabled patients to identify previously overlooked COPD-related issues, thereby preparing them to articulate their recognized psychosocial challenges and concerns. None of the patients mentioned any existential issues.

Responding to each question in PRO-Pall stimulated the patients to reflect upon their current situation. They explained, that the completion of PRO-pall prompted them to: “*…think about..,”* “*…have thoughts about…,”* or “*…get their thoughts going on…”* (P1–P4, and P6) regarding their daily challenges and concerns related to COPD. Two patients expressed that completing PRO-Pall did not trigger any subsequent reflection before the consultation:

- “I just filled it (PRO-Pall) out, you know, and yeah, it was easy enough, and then I didn’t really think about it anymore.” (P9)
- “No, not really, but of course, one couldn't help but think about how bad one feels sometimes. And then, of course, when one got into that conversation about it at the hospital.” (P4)

However, all patients experienced some level of reflection before the consultation, although it appeared that not all patients were aware of this. Patients noted that completing PRO-Pall prepared them for the outpatient consultation by providing an opportunity to reconsider their current situation more profoundly. One patient expressed this as follows:

- “I kind of thought it through a bit better. You know, because you think about it and look back when you have to answer those questions. Well, actually, I think the questionnaire hit the nail on the head; it caught what you’re struggling with.” (P1)

Most patients emphasized the significance of this reflection in fostering awareness and gaining new insights into their situation and the impact of COPD on their well-being. Specific questions addressing psychosocial aspects were perceived as particularly probing and uncovered concerns and issues that they had not previously contemplated or recognized. One patient encountered difficulty in responding to specific questions, especially the one about missing intimacy. The challenge was not in understanding the question, but rather in dealing with its personal nature, as it addressed an issue he had never previously considered or acknowledged. Nevertheless, he realized the importance of uncovering this sensitive and overlooked aspect:

- “Yeah, I remember sometimes thinking: they’re getting a bit close, but nothing happens with that. I’m an old man and I can handle answering (laughs a bit). So I did. (…) I thought, “what does it really have to do with my lungs?” But yeah, I can see that if this and that. Well, then they do have something to do with each other. So, I came to the realization, that it had something to do with my COPD.” (P3)

The patients highlighted four specific items in particular that prompted them to reflect on and uncover previously overlooked issues. These items addressed concerns related to intimacy, loneliness, depression, and changes in their role with family or friends.

Patients expressed in various ways how completing PRO-Pall stimulated them to recall and consider their situation more profoundly. One patient viewed the PRO-Pall as a necessary “*push”* that facilitated articulation of her thoughts and prepared her to discuss her feelings and concerns (P7). Another patient emphasized the significance of his reflection and awareness, stating:

- “When the nurse started asking those questions, you had kind of been thinking about it, and then it was easier to explain. In that way, it was good. (..) It's a bit like an exam: you get a push, so you think a bit more about it. I probably wouldn’t have done that without it (PRO-Pall). So, I was kind of more ready for the talk.” (P2)

Most of the patients perceived that the reflective process “*made it easier”* for them to articulate and explain their perspectives, leading them to feel more “*prepared”* or “*ready”* to express their thoughts and challenges to HCPs.

#### 3.2.2 Theme 2. Unmasking concerns: completing PRO-Pall encouraged patients to embrace honesty rather than concealing concerns

Completing PRO-Pall encouraged most patients to confront their inner truths and practice self-honesty. This led them to refrain from concealing their psychosocial matters, which prepared them to unveil their challenges and concerns during their subsequent consultation.

Patients described this process of unmasking in various ways, yet the majority perceived it as a realization, through which they consciously acknowledged their previous tendency to conceal or sugarcoat certain feelings or concerns to avoid discussing sensitive or difficult topics. Reflecting on their responses, one patient expressed that he deliberately chose to respond truthfully to all questions upon realizing that withholding information would not serve his best interests:

- “But I can say that I’ve laid all my cards on the table and answered as I felt. So, there's nothing hidden in those responses. But I must honestly admit I had thoughts at first, but then I realized that it had something to do with my COPD and that I wouldn’t gain anything from hiding it.” (P3)

Several patients described that they consciously chose to reveal concerns, as they realized that sugarcoating or concealing their emotions and concerns was counterproductive. Nevertheless, they found their concerns challenging to describe. Four patients used the expression “*taking off the mask”* to explain how the PRO-Pall completion encouraged them to answer questions honestly and prepared them to engage in authentic discussions about their emotions and concerns. For example, one patient expressed:

- “I’m one of those who puts on a clown mask when someone asks, “How are you doing?” It's because, many times, if I start answering, I’ll start crying. And I definitely don’t want to do that in front of anyone. You know, in a way, it's like undressing — admitting how miserable you actually are. (…) Now, it was easier to take off the mask and be a bit more open than I usually am. Because I’m not one of those who blurt out all my miseries.” (P7)

Several other patients had previously hesitated to be honest and open about specific challenges and concerns, either due to a perceived lack of relevance or a personal inclination to keep certain emotional aspects private. However, once prompted by completing PRO-Pall, they were somehow encouraged to confront their inner truths and embrace honesty by answering truthfully. This prepared them to openly unveil their genuine emotions and concerns during subsequent consultations. One patient elaborated on his adjusted strategy:

- “It was like an “aha moment” — you know, where it became more apparent to me that I can’t just keep it to myself.. Because I had marked some crosses, so I couldn’t really run away from that. (..) I mean, the thing about feeling depressed, and having these thoughts that I’m not good at anything and feeling sad and tired and all that. It's not something I usually blurt out.” (P2)

Some patients characterized these experiences as moments of realization of truths that highlighted the ineffectiveness of sugarcoating or concealing their emotions and concerns. This change in approach was identified among most patients, as they tended to shift from avoidance or pretense to being honest and prepared to unveil their concerns candidly. While most patients reported honestly, two patients admitted to sugarcoating their responses, either due to discomfort or unfamiliarity discussing psychosocial concerns openly. They were used to keeping such matters to themselves. Nonetheless, they believed that completing PRO-Pall had prompted some thoughts, making them ready to disclose and discuss these matters when addressed during consultation. Two patients still appeared to consciously choose to conceal their emotions and concerns when completing PRO-Pall. One of them expressed hesitancy in reporting feelings of being depressed during the research interview:

- “It's fine, but if I were sad, I don’t know if I would have answered that I’m outright depressed. You can’t fix that anyway. It's not something I feel I should bring up at the hospital. All that emotional stuff, I don’t want to. No, it's just not my thing. (…) I’ve sort of chosen this myself, so I can’t complain, I could just stop smoking.” (P5)

This implies that for some patients, completing PRO-Pall alone is insufficient, and that their self-blame further contributes to their tendency to conceal concerns. Another patient sought her daughter's assistance in answering the questions, acknowledging beforehand that she might not have responded entirely truthfully otherwise. In this instance, it was the daughter's inquiries that prompted her to embrace honesty:

- “Well, some of the questions were difficult, right? Because we got to the point that says, “Are you depressed?” So I said, “No, I’m not.” Then she (the daughter) said, “Are you sure about that?” And then I realized, “Well, I am depressed about what I can’t do anymore.” (…) But I’ve started on it now, and I no longer sugarcoat it. So, it's only now, after the questionnaire, that I see that it doesn’t help to sugarcoat it. It was like I became ready to stop sugarcoating it—that is, if they asked.” (P6)

The final statement “*if they asked”* implies that the patient might still conceal her feeling of being depressed if it is not addressed by the HPCs. The majority of patients did not have a clear expectation of how their responses would be utilized; however, all patients expressed an expectation that the HCPs would address their responses in some way during consultations. One patient found comfort in knowing that completing PRO-Pall provided the HCPs with insight into his experiences, thereby reducing the urgency for him to recall and mention important issues during the consultation. This seemed to prompt him to answer questions honestly:

- “It was fine for me, you know, with having to check off here and there for the different questions and stuff like that. There are many things you forget when you’re in it (the consultation). So, it sort of helps to cover everything. Yeah, and it's also good to know that they (HCPs) kind of know what's important. So, in that way, it's also nice.” (P1)

Similarly, several other patients mentioned their memory difficulties as reasons for not accurately describing their thoughts during PRO-Pall completion by stating: *“I can't remember…”* (P3, P4, P5, P6, P7 and P8). Overall, the patients believed that their responses were helpful for the HCPs, and this belief appeared to encourage them to respond honestly and to reveal their perceived challenges and concerns, although not all patients were willing or prepared to do so.

#### 3.2.3 Theme 3. Breaking the ice: Hcps’ direct questions initiated discussions about sensitive yet vital matters

All patients experienced that their PRO-Pall responses enabled the HCPs to inquire directly about identified issues. For most patients, this was crucial for legitimizing and initiating discussions, particularly centered on previously overlooked psychosocial challenges and concerns. These issues were perceived as sensitive yet vital due to their notable impact on patients’ well-being.

The patients were generally very satisfied with the proactive use of their PRO responses by the HCPs. They described how, during the initial consultation, the nurse familiarized herself with their responses, using them to ask direct questions about identified issues. This then opened up discussions about the patients’ current challenges and concerns:

- “She went through them—the various things I had marked—and then we talked about it. (…) We discussed the different questions because she inquired about them.” (P5)
- “She addressed it and was not afraid to ask about those things that might be a bit difficult (…). She came straight to the point and said, “Now you answered like this and that, what do you mean by that?” Well, I thought she did a really good job.” (P3)

Several patients expressed uncertainty about how or whether the physician utilized their PRO-Pall responses. However, they perceived this as less significant, as the nurse had already addressed identified issues. Some patients observed that the nurse passed on selected information to the physician, allowing a continuation of the conversation that often focused on medical or treatment-oriented aspects, as exemplified in the following statement:

- “… Some (questions) we talked more about, and then she (the nurse) passed on some information to the doctor. The doctor continued the conversation from where she left off, you could say. (…) With the doctor, it was probably more about the lungs and how we can improve them, with medications and stuff.” (P1)

The patients used various expressions when describing, how their PRO-Pall responses impacted the consultation. Four patients used the term “*breaking the ice”*, signifying that is was the direct questions that made it “*easier to open up”* or possible to discuss personal or difficult topics (P1–P3, and P7). For instance, some patients noted that the direct questions “*got the conversation started”* (P3) or “*opened up for those talks”* (P6) about “*…what was important,”* “*…what was difficult,”* “*…the difficult things,”* or “*…the things that bothered.”* Additionally, one patient felt that the direct questions made him think, “*It was like they knew me”* (P1). These statements underscored the patients’ perception of psychosocial issues as being sensitive and difficult yet vital to address and discuss, with the direct questions playing a pivotal role in initiating these discussions. Two patients explained how they perceived that the direct questions helped them:

- “…to get hold of it and to break the ice on what's difficult and that you might not have the courage to burst out with on your own.” (P7)
- “They (the staff) knew more about it in advance, so they could ask about it more directly, without beating around the bush. Then we could talk about things that bothered me—the difficult but important things.” (P4)

The quotes illustrate the difficulty they had in discussing certain concerns while also emphasizing the importance of addressing these sensitive topics. Most patients felt more comfortable discussing their challenges, because the HCPs had prior knowledge obtained through PRO-Pall. They attributed this new openness to the direct questions from the HPCs, which encouraged the patients to unveil psychosocial challenges and concerns that they had never previously discussed with HCPs or relatives. This was notably evident in one patient who specified that it was the first time he had talked with anyone about his sexual problems:

- “…that we kind of broke the ice on the talk about my sex life. (..) I said that because they asked about my mark on that questionnaire.” (P1)

Despite the direct questions, two patients did not engage in honest discussions on psychosocial issues. One patient, who had indicated missing intimacy in PRO-Pall, refrained from discussing this issue due to the presence of his spouse and her dissenting comments, which effectively ended the discussion on this sensitive topic. The other patient explained that he only needed to discuss his lung function, medications, and his need for ergonomic aids, yet he expressed: “*It's definitely important to talk about it afterward.”* (P8). Nonetheless, with few exceptions, the patients openly discussed feelings of loneliness, sadness, inadequacy, defeat, anger, or intimacy issues for the first time.

#### 3.2.4 Theme 4. Deepening the dialogue: HCPs’ targeted and attentive approach fostered meaningful discussions and deeper understanding

Overall, the patients valued the HCPs’ proactive and attentive use of their PRO-Pall responses, which broadened consultations to encompass not only physical symptoms but also COPD-related psychosocial issues. Most patients emphasized the importance of initial discussions being targeted on their current issues, as it fostered a more comprehensive exploration of their challenges and underlying concerns. Patients expressed gaining a new or deeper understanding of the psychosocial burden of their COPD, and therefore perceiving consultations as more meaningful than their previous experiences with clinical consultations.

Patients perceived that the HCPs’ utilization of their PRO-Pall responses fostered more comprehensive discussions on individual matters, and most patients felt that they were able to “get around,” “through,” or “cover” everything during consultations (P1–P3, P6, P7, and P9). Many patients were pleasantly surprised by the HCPs’ attentive interest and dedication of time to listen to their perspectives. Several patients perceived that the targeted approach, facilitated by their PRO-Pall responses, streamlined consultations and provided more time to engage in deeper discussions on their most challenging or concerning matters. The patients observed and valued that HCPs invested time in exploring not only the physical symptoms but also the psychosocial aspects affected by the condition, which also had an impact on their well-being. One patient expressed it as follows:

- “Yeah, and nice to feel that they have time for you and all that. I think they see you as a whole person. It's not just the lungs, right? They can handle hearing about the side effects it brings. I think that's good. It (the illness) puts limitations on everything.” (P6)

This quote illustrates how most patients seemed unfamiliar with consultations that delved into their concerns beyond physical symptoms. Furthermore, it highlights that patients valued being seen and met as individuals. Most patients noted the HCPs’ attentive approach, as they felt listened to. They valued the opportunity to explore and discuss not only their physical issues, but also their psychosocial challenges and concerns. Some found that this fostered openness and trust.

Additionally, all patients except one expressed that the consultations, particularly the initial dialogue with the nurse, were either more comprehensive or more profound than they were used to. They valued that the HCPs’ use of their PRO-Pall responses as a means to inquire about patients’ perceived challenges and to delve into their underlying causes:

- “We talked about a lot of things—everything that one struggles with. It (the conversation) could sort of start where the shoes pinch, you know. So, I said that I felt depressed and carried these thoughts that I’m not good at anything, felt sad and tired and such. What we talked about was mostly about how I struggle to keep my spirits up, you know. (…) You kind of discuss it more thoroughly because you’re asked more in-depth about what lies beneath. I mean, why I was tired and tense? You get further into the conversation.” (P2)

The patients perceived that the HPCs’ proactive utilization of their PRO-Pall responses fostered deeper and more comprehensive communication about previously unaddressed challenges and concerns. One patient, upon reflecting on the usefulness of PRO-Pall, recounted how he came to a new understanding during the consultation. For the first time, he recognized that his feelings of depression were related to or caused by his erectile dysfunction and lack of sexual intimacy:

- “It's the lungs, but it's also an erectile problem. Both things, really. Because if it were just an erection issue, I might think it's not too bad, but when we get started, I can’t breathe either. So, it just won’t work anyway, you know. And then you’re faced with another failure, and you think, “No, there's no point in starting because I can’t complete it.” And, actually, I believe it's the reason for the depression I probably have.” (P3)

Similarly, another patient described how she recognized an unhelpful avoidance behavior:

- “And, like, when I’m out, I gotta remember that sometimes (during the walk) I gotta speak up to the family when they invite me somewhere. I talked to the nurse about that. It's because sometimes I fall behind and I don't think it's cool for the family to have to wait for me. It's kinda like a defeat, so it's not cool. But then, like, I gotta say it and not just opt out. And I talked to my son about it afterward, ‘cause he didn't know anything about it.” (P9)

Several patients came to realize that their breathlessness often led them to avoid activities such as cooking, dancing, or taking walks with their relatives, which had a negative effect on their mood. Subsequently, some patients shared their newfound insights or deeper understanding with their spouses or families, expressing that it was an important first step towards improving their well-being.

## 4 Discussion

To our knowledge, this is the first study to investigate how patients with COPD experience and perceive the utilization of a holistic PROM before and during outpatient consultations. The following sections will summarize and discuss our findings, structured according to our two study objectives.

### 4.1 Completing PRO-Pall before consultation

Completing PRO-Pall before consultations stimulated the majority of patients to reflect on their COPD-related physical and psychosocial issues. This enabled the patients to recognize psychosocial challenges and encouraged them to refrain from concealing their concerns.

Our findings align with previous research emphasizing the benefits of PROM completion in facilitating patients’ self-reflection and enhancing their self-awareness (61, 62, 16). When patients gain insights into the aspects of daily living that are affected by COPD, this can potentially promote patients’ adherence to and satisfaction with treatment (63). Our study also revealed that completing PRO-Pall encouraged most patients to candidly confront their psychosocial challenges and concerns, thereby preparing them to articulate and disclose these previously overlooked issues. However, several studies have shown divergent levels of honesty among patients who complete PROMs. While some patients reported increased honesty, others felt that the PROM facilitated dishonesty, possibly due to the specific content of the PROM (61). Patients may choose to conceal stigma-related issues, such as depression, to avoid judgment from HCPs or unwanted medical treatment or psychological assistance (64). Nevertheless, using PROMs seems to facilitate discussions on difficult or sensitive topics, like sexual or mental health issues (61).

We also found that some patients had difficulty in expressing concerns, such as depression, loneliness, and intimacy, and often hesitated to disclose them, as they consided them sensitive and unfamiliar, and yet important, topics to discuss. This reluctance to disclose certain issues is supported by previous studies that have shown that although patients often worry about COPD progression and end-of-life care, they hesitate to express these concerns. This hesitation may stem from a lack of awareness or understanding (65, 66). Patients with palliative care needs, including those with advanced COPD, prefer open and honest communication, with HCPs proactively initiating discussions on individual needs and concerns (10, 66). Our results suggest that the utilization of PRO-Pall might serve as a facilitator to reveal patients’ concerns.

Previous studies have suggested that individuals with COPD-related stigmas are more inclined to conceal their condition (67, 68). This concealment strategy may stem from feelings of self-blame, shame, and unworthiness for treatment, as COPD is often perceived as a self-inflicted condition (69–71). Patients may deny or hide breathlessness as a way to avoid embarrassment or to cope with a perceived lack of understanding by others (72). This tendency extends to other chronic conditions, with individuals resorting to concealment of issues such as loneliness (73), mental illness (74), or chronic pain (75).

### 4.2 Discussing PRO-Pall responses during consultation

Discussing PRO-Pall responses with HCPs during consultations appeared to be essential for fostering comprehensive communication targeting identified challenges. Patients revealed how their PRO-Pall responses enabled HCPs to address individual challenges directly and attentively, which was a crucial step in initiating discussions about sensitive, yet vital COPD-related concerns. This fostered a deeper level of communication and understanding of the psychosocial burden of COPD.

Studies have suggested that utilizing PROMs supports patients in expressing their issues through numerical ratings, which may be easier for some patients than describing them verbally (61). While this might be the case in our study, none of the patients mentioned it. During consultations, patients often convey underlying concerns or emotional distress through indirect or nonverbal cues, which physicians may frequently overlook (76). This tendency is notable among patients with COPD and limited health literacy, who often refrain from directly expressing concerns or unpleasant emotions (77).

Limited health literacy is prevalent among patients with COPD, and may hinder self-management and medical adherence, as it impacts the patients’ ability to understand health information and engage in clinical communication (78–80). Furthermore, it is associated with limited health status and quality of life (81–83). Open communication is vital for promoting personalized care and reducing health inequality (84). It is widely recognized that patients perceive PROM utilization as assisting in prioritizing needs and identifying issues that may otherwise have been overlooked (61). Consistent with our results, previous research highlight that the HCPs’ direct inquiries serve as a crucial “icebreaker” and represent one of the key benefits of PRO utilization (85). Our findings also revealed that the majority of patients openly discussed concerns, including feelings of inadequacy, loneliness, depression, anger, and guilt, for the first time. This appeared to be essential for them in gaining a new understanding of the multifaceted burden of COPD. Another important point to note is that most patients in our sample had limited educational backgrounds. This underscores the potential benefits of utilizing PRO-Pall among patients with COPD and/or limited health literacy. Nevertheless, ensuring their capability to complete PRO-Pall is essential. Studies have indicated that health literacy is a potential barrier to PROM completion (20, 62).

Previous research on patient perspectives has demonstrated the potential benefits of PROM utilization, such as enhanced patient engagement and personalized communication and care (61, 62). Patients report that its use fosters discussions on broader issues and concerns, thus facilitating consultations that extend beyond the medical condition (61). These findings align with our study, as our patients consistently expressed ongoing interest in assessing physical issues and the clinical parameter FEV1, which they used as a metric for evaluating the stability of their COPD. Nevertheless, several patients were pleasantly surprised by their HCPs’ interest in all aspects of daily living that were affected by COPD. This suggests that PRO-Pall meets its intended purpose by fostering holistic communication, as it was developed to address the whole patient and palliative care needs with a holistic approach (33). We found that PRO-Pall was relevant and useful for patients who differed in sex, age, and COPD severity, even those who may be far from the terminal phase. Patients with COPD frequently have unmet care needs that required a palliative or holistic approach to address needs related to physical, psychosocial, informational, and practical aspects (86). Hence, HCPs should proactively initiate holistic discussions to prevent patients from resorting to concealing their concerns and potentially compromising their well-being (72). Our findings suggest that the usefulness of PRO-Pall hinges on whether or not the HCPs’ utilize patients’ responses to address identified issues.

When the HPCs are reluctant to address holistic challenges, it could hinder the patients’ disclosure of concerns and open communication (69, 72). Some HCPs perceive emotional distress, sexual health, or existential issues as irrelevant or beyond their scope, despite patients’ wishes to discuss these concerns (16, 87–89). Existential issues were not addressed in our study. It remains unclear whether this is because the single existential item in PRO-Pall did not prompt existential considerations or because patients did not experience any existential issues. This might only be the case among patients who are newly diagnosed with severe COPD or those who have mild or moderate COPD (89).

Open and comprehensive communication can also be hindered by time pressure during consultations (72). In our study, patients consistently emphasized the importance of encountering an empathetic, engaged, and attentive HCP who devoted time to listening to their perspectives. Our positive outcomes might be partly attributed to organizational strategies, such as our dedicated and experienced PRO-Pall team. Effective utilization of PROMs requires that HCPs not only interpret and integrate PROM responses into routine practice (20), but also focus the communication on patients’ individual challenges and concerns (61, 10, 90). Additionally, the HCPs should embody interpersonal qualities, such as courage, humility, curiosity, and flexibility, to fully leverage PRO-Pall's potential in promoting person-centered care (91).

Our results indicate that integrating PRO-Pall into outpatient consultations among patients with COPD can facilitate holistic communication, ultimately enhancing patients’ well-being. However, further research is required to explore: (1) the benefits and limitations of PRO-Pall utilization among patients with limited health literacy at different stages of the patient trajectory and across various healthcare settings, (2) the impact of integrating PRO-Pall into routine outpatient COPD care on patients’ longitudinal COPD management and well-being, (3) whether PRO-Pall is useful to assess existential issues, and (4) how PROMs in general can promote involvement of relatives in person-centered consultations.

Exploring the usefulness of the PRO-Pall in clinical practice is essential. For instance, it is important to evaluate whether PRO-Pall not only improves patient participation during consultations (92), but also contributes to reducing patients’ perceived health-related stigma (93), and enhancing their psychosocial well-being (94). Addressing these broader objectives will provide valuable insights into the full scope of PRO-Pall's potential benefits. Applying quantitative or qualitative methods tailored to these objectives will be crucial for assessing the effectiveness of PRO-Pall and ensuring it meets its intended goals.

### 4.3 Strengths and limitations

To our knowledge, this study is a pioneering qualitative investigation into patient experiences with the utilization of a holistic PROM to facilitate person-centered consultations among outpatients with COPD, filling a gap in the current literature. A primary strength lies in our comprehensive approach, capturing the perspectives of fragile individuals facing stigma and limited health literacy, whose daily challenges are often overlooked. The depth and richness of our data enabled the development of a conceptual model illustrating the utility of PROM utilization in fostering honest and meaningful consultation.

Despite these strengths, our study has limitations. First, its confinement to a specific holistic PROM among stable patients with COPD within a single respiratory clinic reduces the transferability of the findings to more diverse patient populations. Nevertheless, aligning our findings with previous research on PRO utilization underscores their potential applicability to similar settings and patient populations (51). Second, potential selection bias may exist, as the participants were all recruited by the same PRO-Pall coordinator. Despite efforts to use flowchart guidance to ensure diversity in participant selection, less engaged or satisfied patients may have been excluded. Sensitivity related to reported outcomes may also have deterred some patients from participating. Third, conducting the initial coding and analysis without co-researchers could potentially have impacted our interpretation. However, our use of predefined research questions and documentation in a coding tree enhanced the comprehensiveness and transparency of the data interpretation (52, 95).

## 5 Conclusion

Completing a holistic PROM for basic palliative care (PRO-Pall) before consultations in a respiratory outpatient clinic, as highlighted in our interview study, stimulated patients to reflect more profoundly on their COPD-related issues, thereby uncovering previously unacknowledged psychosocial challenges and preparing them to reveal and discuss their concerns. The patients also perceived that the HCPs’ proactive use of PRO-Pall responses during consultations played a crucial role in promoting a targeted and attentive approach that fostered more person-centered and meaningful discussions about sensitive yet important psychosocial challenges and concerns, many of which patients had never previously discussed. This approach appears to provide most patients with a deeper level of understanding of the multifaceted burden of COPD. Nevertheless, some patients remained reluctant to reveal their concerns.

## Data Availability

The datasets presented in this article are not publicly available due to confidentiality concerns. Participants consented only to the publication of excerpts and non-identifiable results. Given the specific time period of data collection and the outpatient clinic involved, there is a risk that patients could potentially be identified if the raw data were shared. However, access to the data may be granted upon request for legitimate research purposes. Requests for access should be directed to louise.muxoll.gronhaug@rsyd.dk.
