# Supplementary material for: Unveiling the burden of COPD: perspectives on a patient-reported outcome measure to support communication in outpatient consultations—an interview study among patients
Source: Front Rehabil Sci. 2024 Sep 2;5:1434298. doi: 10.3389/fresc.2024.1434298 (PMC11421387; doi:10.3389/fresc.2024.1434298)
Supplement: Supplementary file 1 [file Datasheet1.pdf]

Supplementary Material

Article Title:

Unveiling the burden of COPD: Patient perspectives on a Patient-Reported Outcome Measure to support communication in outpatient consultations

Louise Muxoll Gronhaug[12*], Ingeborg Farver-Vestergaard[12], Jannie Christina Frølund[1], Cecilie Lindström Egholm[34], Anders Løkke Ottesen[12]

*** Correspondence:** Louise Muxoll Gronhaug, louise.muxoll.gronhaug@rsyd.dk

The interview guide

The guide is designed to investigate two research questions:

1) How does completing PRO-Pall before consultations prepare patients to engage in discussions about their COPD-related issues and concerns with HCPs?
   - Based on the presented literature (Background), we assume that when patients complete PRO-Pall before the consultation, they are likely to feel better prepared to engage in discussions about their current COPD-related issues and concerns with healthcare providers. Additionally, we assume that patients will report a range of issues, including physical, psychosocial, and existential aspects.
2) How are PRO-Pall responses utilized during consultations, and how does it contribute to discussing patients' individual COPD-related issues and concerns?
   - We assume that patients will experience that the healthcare providers are using their PRO-Pall responses to open and facilitate communication centered on identified issues across both physical, psychosocial, and existential aspects affected of their COPD.

**Introduction**
Brief introduction to the:<br>- Interviewer: research nurse - not regularly present in the clinic - familiar with the workflow<br>- Study: background, overall aim, and method
**The individual with COPD**
We are going to talk about your experience with your recent visit to the Lung clinic and what you thought about using the questionnaire "How Are You Feeling?" (PRO-Pall).<br>But before that, I would like to hear a bit more about you and your daily life.

<table>
<tr><td>Background information</td><td>- So, can you tell me a bit about yourself?
    o What is your age, education, work and living situation?
- How many years have you had COPD?
    o If you had your lung function measured at the Clinic the other day, what was it?
- When was the last time you were hospitalized?</td></tr>
<tr><td colspan="2">1. Before consultation: Completion of PRO-Pall as preparation</td></tr>
<tr><td colspan="2">I'd like to hear about your experiences with filling out the "How Are You Feeling" questionnaire (which is what PRO-Pall is called among patients).</td></tr>
<tr><td>Observation</td><td>- What information had you received about the questionnaire?
- When and where did you answer the questionnaire?</td></tr>
<tr><td>Experience</td><td>- How was it to answer the questions?
    o What thoughts or feelings did the questionnaire evoke in you?
- What did you expect your answers to be used for?</td></tr>
<tr><td>Interpretation</td><td>- Can you describe what impact it had for you to fill out the questionnaire?
    o How did it make you feel prepared for the consultation?</td></tr>
<tr><td colspan="2">2. During consultation: Use of PRO-Pall responses to discuss individual issues</td></tr>
<tr><td colspan="2">Now, try to think back to the first conversation you had with the nurse.</td></tr>
<tr><td>Observation</td><td>- Who was present during the conversation?
- Try to describe what happened
    o What did the nurse say or do?
- How did the nurse use your answers from the questionnaire?
    o How did the nurse make sure to talk about the things that were difficult or important to you?
- Now think about the conversation you had with the doctor. Can you tell me about that?
    o What did the doctor say or do?
- How did the doctor use your answers from the questionnaire?
    o What did the doctor do to talk about the most difficult or important things?</td></tr>
<tr><td>Experience</td><td>- What do you feel the questionnaire added to the discussions?
    o How did you experience that you got to discuss what you needed to talk about?
- What's bothering you the most right now in relation to your COPD?
    o In what way did the questionnaire contribute to discussing this?</td></tr>
<tr><td>Interpretation</td><td>- What impact did it have on you that the nurse or doctor (did not) address the things you had noted as difficult or problematic in the questionnaire?
- Overall, how would you rate the conversations in the outpatient clinic?
    o What was the main insight or takeaway from the consultations for you?
- What do you think about having to fill out the questionnaire again at your next visit?</td></tr>
</table>

Final thoughts	
Suggestions	- Did you have any unaddressed questions or concerns during your visit to the Lung Clinic?<br>- Was there something you would have preferred differently?<br>o related to the questionnaire, the conversations, or anything else?
Closing the interview	- Do you have anything else on your mind that you haven't said but think is important to express before we finish?<br>- How did you experience participating in the interview today?
