# Supplementary material for: Unveiling the burden of COPD: perspectives on a patient-reported outcome measure to support communication in outpatient consultations—an interview study among patients
Source: Front Rehabil Sci. 2024 Sep 2;5:1434298. doi: 10.3389/fresc.2024.1434298 (PMC11421387; doi:10.3389/fresc.2024.1434298)

Supplementary Material

Article Title:

Unveiling the burden of COPD: Patient perspectives on a Patient-Reported Outcome Measure to support communication in outpatient consultations

Louise Muxoll Gronhaug[12*], Ingeborg Farver-Vestergaard[12], Jannie Christina Frølund[1], Cecilie Lindström Egholm[34], Anders Løkke Ottesen[12]

*** Correspondence:** Louise Muxoll Gronhaug, louise.muxoll.gronhaug@rsyd.dk

The analytic steps and coding tree

The interview texts and field notes were analyzed using qualitative content analysis. This process was conducted inductively as an iterative process, consisting of four steps:

1) **Establishing an overview of the material**
   - Initially, the data material (transcripts and field notes) of the first five interviews were thoroughly read by the interviewer to familiarize with the manifest content of the text.
   - This process was repeated after each subsequent interview to evaluate if there was sufficient data to address the two study objectives, which were determined to be fulfilled after the ninth interview.
   - Subsequently, the interviewer read the entire dataset to establish an overview.

2) **Identifying and extracting meaning units with research questions**
   - The initial coding scheme was developed from the first five interviews and continuously refined thereafter.
   - Meaning units were identified and labeled within the NVivo 14 software using a text-based, one-word code, guided by the study objectives and assumptions.
     Only units relevant to study objectives were extracted for further analysis, while text units deemed not relevant were labeled as “other” and excluded from the analysis.
   - To ensure that important wording (content) was not overlooked, a "word frequency query" was conducted to display the most frequent words as Tree Maps and Word Clouds
     For example, the following Word Cloud includes the 60 most frequent meaningful words (stemmed words with ≥ 6 letters and excluding stop words like become, otherwise, right.
     The 10 most frequent words were: nurse (sygeplejersken), how (hvordan), thinks (tænker), questionnaire (skemaet), completing (udfyldte), actually (egentlig), talking (snakke), around (omkring), thought (tænkte), asked (spurgte).

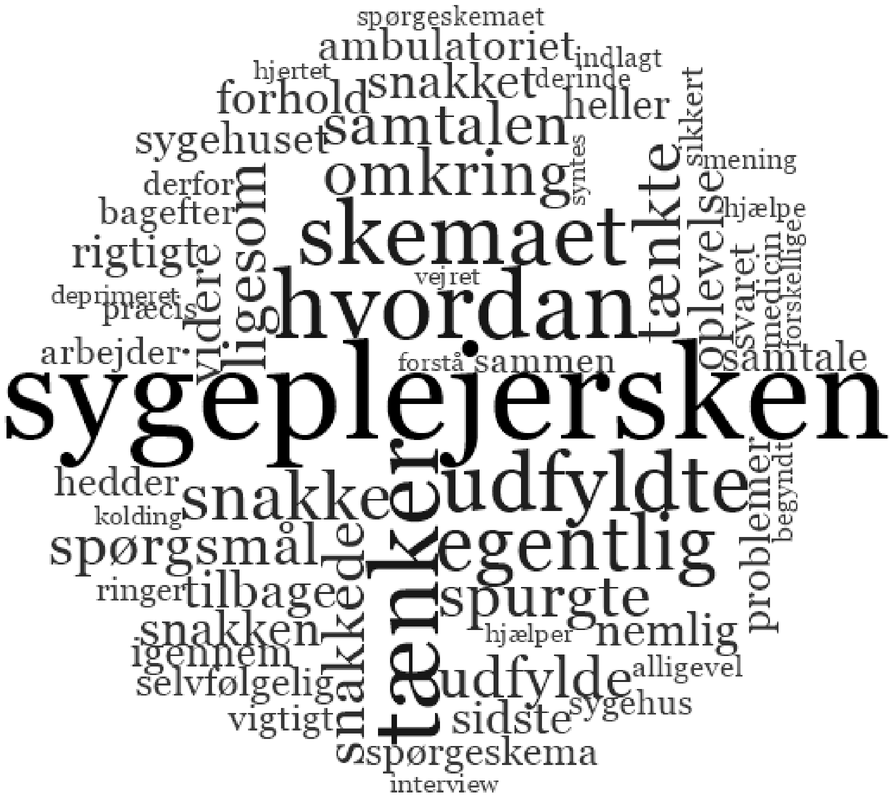

3) Condensating meaning units into descriptive categories

- Then, the first author conducted the comparative analysis of the extracted meaning units, focusing on distinguishing similarities and differences within and across codes. Meaning units sharing common attributes, including similar or contradictory content, were clustered together and subsequently condensed. The analysis focused on resemblances in meaning, resulting in 22 descriptive categories.
- NVivo was utilized to uncover overlaps and reveal relationships between codes by conducting complex queries and generating comparison diagrams. For example, the codes “hide” and “sugarcoat” were merged into “concealment” (of challenges or concerns) as they shared the same latent meaning. The relationship (association) between the codes “concealment” and “honesty” was explored by generating a comparison diagram. The comparison diagram below is extracted from NVivo, where participants are named P1-P9. It illustrated and revealed that most patients (P2, P3, P5, P6, P7, P9) were coded as both “concealment” and “honesty”, which prompted further analysis and thus contributed to the generation of a category.
-

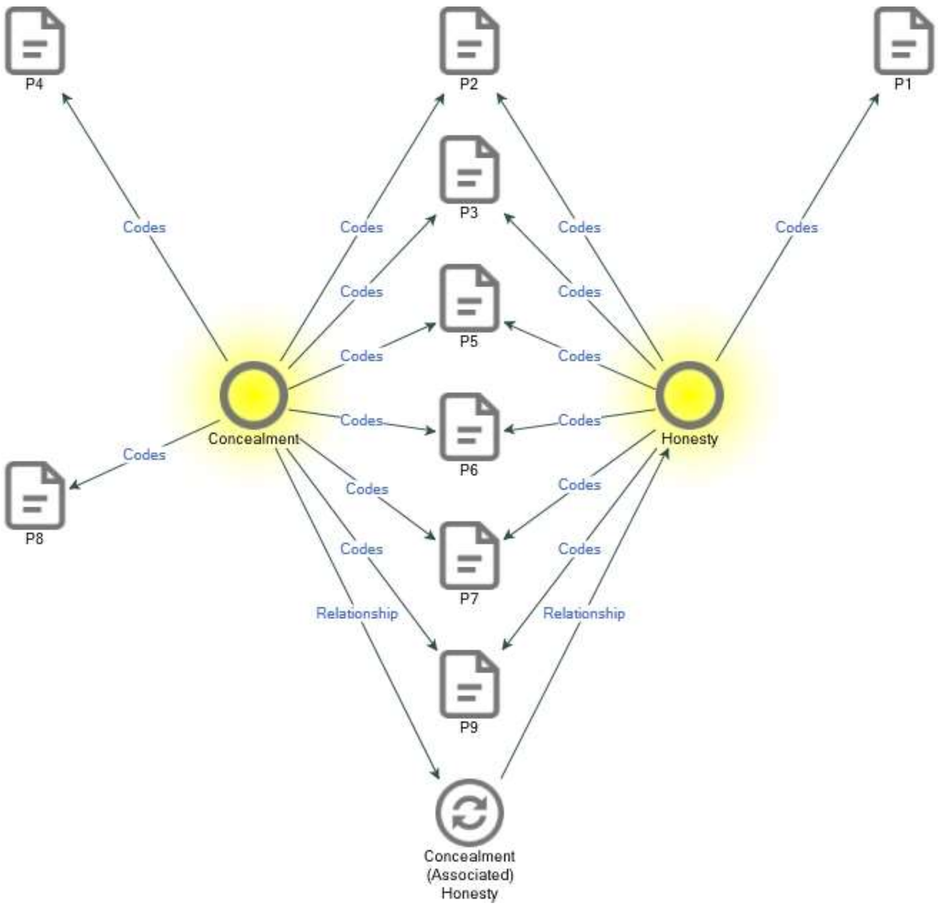

- The coding scheme was adjusted as needed, with ongoing refinement, which included renaming some codes and sub-categories. This adjustment necessitated the re-coding of previous interview texts (back to step 2).
  Descriptions of sub-categories and categories evolved gradually with increasing levels of abstraction and interpretation. Examples of meaning condensation and categorizing are presented in the table below.

Meaning unit (quote)	Condensed meaning unit	Sub-categories	Category
Before consultation			
”Yes, but I've started on it now, not to sugarcoat it anymore. I mean, it's only now after the questionnaire, I could see that it doesn't help to sugarcoat it anymore, feeling depressed." (P6)	Acknowledging previous sugarcoating, yet beginning to be honest prompted by PRO-Pall.	Honesty (being truly honest)<br><br>Concealment (sugarcoat)	Embracing honesty rather than concealment

During consultation			
”...because we sort of opened up a theme that, well, I couldn't quite see myself how to approach or find an opening for that question concerning my sex life” (P3)	Experiencing HPC opened up dialogue on a difficult topic	Initiate dialogue on overlooked issues<br><br>Legitimize discussions on sensitive topics	Initiating discussions on sensitive challenges

4) Generating explanatory themes

- Comparing the categorized meaning units led to the identification of meaningful patterns, resulting in the generation of four overarching themes that captured the latent content and provided a comprehensive understanding of the impact of PRO-Pall utilization in outpatient consultation.
- To enhance the validity and reliability of the findings, the preliminary themes were discussed with the second author, who is a psychologist and a researcher in pulmonary and behavioral science, and the third author, who is an expert and researcher in PROMs. Themes were revised based on the consensual understanding and eventually formulated as the final four themes.
- Themes and main findings were further reviewed by the fourth author, a clinical nurse specialist with extensive experience in development and research within the medical department, and by the fifth author, D.M.Sc and Professor in respiratory and cross-sectoral research.
- Final themes and categories were documented in the following coding tree used for analysis in NVivo (highlighted words were utilized as 36 one-word codes).

Coding tree (QCA): Experiences and perception of PRO-Pall utilization among patients with COPD

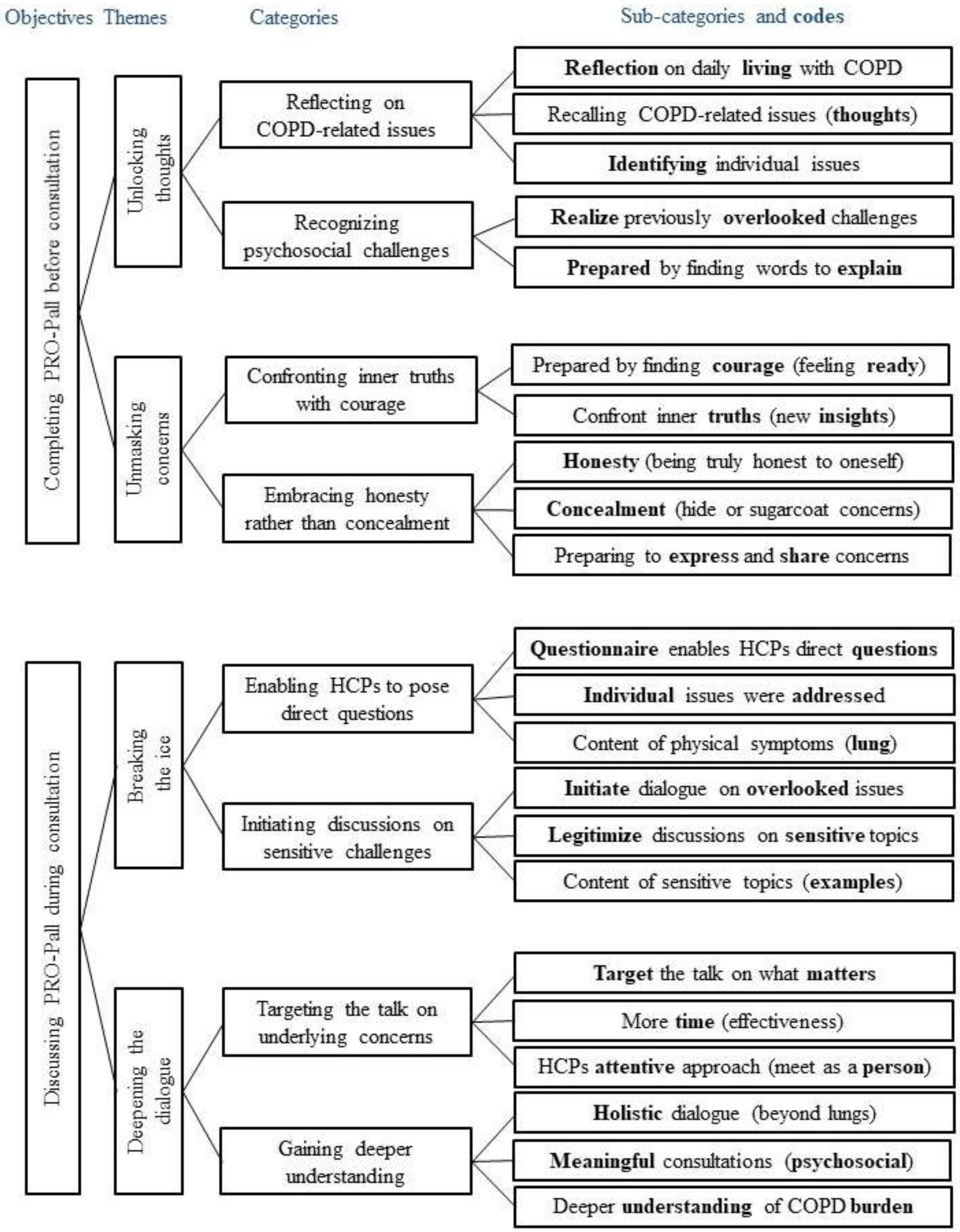

Supplement: Supplementary file 3 [file Datasheet3.pdf]
